# Pharmacological inhibition of Src family kinases attenuates hyperuricemic nephropathy

**DOI:** 10.3389/fphar.2024.1352730

**Published:** 2024-03-21

**Authors:** Chongxiang Xiong, Jin Deng, Xin Wang, Qidi Hou, Shougang Zhuang

**Affiliations:** ^1^ Department of Nephrology, The First Affiliated Hospital of Dongguan, Guangdong Medical University, Dongguan, Guangdong, China; ^2^ Department of Nephrology, The First Affiliated Hospital, Hengyang Medical School, University of South China, Hengyang, China; ^3^ Department of Medicine, Rhode Island Hospital and Brown University School of Medicine, Providence, RI, United States; ^4^ Department of Nephrology, Shanghai East Hospital, Tongji University School of Medicine, Shanghai, China

**Keywords:** hyperuricemia, hyperuricemic nephropathy, Src family kinases, Src, fibrosis, kidney, NF-κB, PP1

## Abstract

Hyperuricemia is an independent risk factor for chronic kidney disease and contributes to renal fibrosis. This study aims to investigate the effect of Src family kinase (SFK) inhibition on the development of hyperuricemic nephropathy (HN) and the mechanisms involved. In a rat model of HN, feeding rats a mixture of adenine and potassium oxonate increased Src phosphorylation, severe glomerular sclerosis, and renal interstitial fibrosis, accompanied by renal dysfunction and increased urine microalbumin excretion. Administration of PP1, a highly selective SFK inhibitor, prevented renal dysfunction, reduced urine microalbumin, and inhibited activation of renal interstitial fibroblasts and expression of extracellular proteins. PP1 treatment also inhibited hyperuricemia-induced activation of the TGF-β1/Smad3, STAT3, ERK1/2, and NF-κB signaling pathways and expression of multiple profibrogenic cytokines/chemokines in the kidney. Furthermore, PP1 treatment significantly reduced serum uric acid levels and xanthine oxidase activity. Thus, blocking Src can attenuate development of HN via a mechanism associated with the suppression of TGF-β1 signaling, inflammation, and uric acid production. The results suggest that Src inhibition might be a promising therapeutic strategy for HN.

## Introduction

Hyperuricemia (HUA) has been reported to be associated with chronic kidney disease (CKD) (Lee et al., 2021). Accumulated evidence demonstrated that the elevated uric acid level contributes to kidney inflammation, tubular injury, tubulointerstitial fibrosis, uric acid, kidney stones, and chronic interstitial nephritis, leading to hyperuricemic nephropathy (HN), CKD, or end-stage renal disease (ESRD) (Xiao et al., 2018). HUA is a common condition that has long been known to have a heritable component. Obesity, diabetes, and chronic kidney failure are conditions with multifactorial inheritance that are associated with HUA (Pan et al., 2019). However, the signaling mechanism by which HUA leads to HN remains unclear.

Src family kinases (SFKs) are a non-receptor protein tyrosine kinase family that regulates a number of biological processes, including proliferation, differentiation, and apoptosis. In humans, the family has 11 members, namely, Src, Fyn, Yes, Blk, Brk (also known as PTK6), Frk (also known as Rak), Fgr, Hck, Lck, Srms (also called Srm), and Lyn. Among them, Src, Fyn, and Yes are expressed in almost all cell types (Brown and Cooper, 1996; Yang et al., 2022; Alrouji et al., 2023). Numerous studies have shown that SFKs are involved in the pathogenesis of various disease processes, including tumorigenesis and tissue fibrosis (Wang and Zhuang, 2017; Tang et al., 2019; Li et al., 2020). Recently, we found that Src is a mediator in renal and peritoneal fibrosis (Yan et al., 2016; Wang et al., 2017). Other studies show that Fyn also plays a role in renal fibrosis (Li et al., 2023) and diabetic nephropathy (Seo et al., 2016). Mechanistically, SFK-mediated tissue fibrosis is related to transformation of renal interstitial fibroblasts into myofibroblasts through activation of the TGF-β1/Smad3, epidermal growth factor receptor (EGFR) and STAT3 signaling pathways, and promoted renal epithelial cells arrested at the G2/M phase of the cell cycle. Src also promoted the macrophage–myofibroblast transition by serving as a direct Smad3 target gene in UUO-induced fibrosis. Although Src expression was reported to be increased in the kidney of HUA animal models (Yan et al., 2016; Xiao et al., 2018; Pan et al., 2019), it is unknown whether Src is involved in the pathogenesis of HN.

In this study, we investigated the role and the mechanism of Src in a rat model of HN induced by the oral administration of adenine and potassium oxonate using PP1, a selective inhibitor for SFKs. PP1 effectively inhibits activation of several SFK isoforms, including Src, Lck, and Fyn (Hanke et al., 1996) and demonstrates an anti-cancer potential for breast cancer and sarcomas in animal models (He et al., 2000; He et al., 2001). Our results indicated that SFKs play a critical role in promoting the pathogenesis of HN through activation of the TGF-β1/Smad3 and NF-κB signaling pathways and expression of multiple profibrogenic cytokines/chemokines in the kidney.

## Methods

### Chemicals and antibodies

Antibodies to p-Src, Src, fibronectin, p-Smad3, Smad3, p-ERK1/2, ERK1/2, p-STAT3, STAT3, p-NF-κB, and NF-κB were purchased from Cell Signaling Technology (Danvers, MA). Antibodies to fibronectin, collagen 1 (A2), and GAPDH tubulin were purchased from Santa Cruz (Santa Cruz, CA). All other chemicals were purchased from Sigma (St. Louis, MO).

### HN model and PP1 treatment

Six 8-week-old male Sprague Dawley rats of weight 220–250 g were purchased from the experimental animal center of Southern Medical University. The rats were housed in a specific pathogen-free (SPF) laboratory at the Animal Experimental Center of Southern Medical University. Experimental protocols were approved by the Southern Medical University Experimental Animal Ethics Committee. The HN rat model was established by oral administration of a mixture of adenine (0.1 g/kg) and potassium oxonate (1.5 g/kg) daily for 3 weeks.

To examine the effects of PP1 on renal fibrosis after HN injury, PP1 (2 mg/kg) in 50 µL of DMSO was immediately administered, i.p., after oral administration of a mixture of adenine and potassium oxonate and then given every other day at the same dose. The selection of this dose of PP1 was according to a previous report (Yan et al., 2016; Xiong et al., 2017). For the HN model-alone group, mice were injected with an equivalent amount of DMSO. Six mice were used in each group. The animals were sacrificed, and the kidneys were removed on day 21 for protein analysis and histological examination. Laboratory animals and animal experiments are reviewed and approved by the Animal Care and Use Committee of Guangdong Medical University.

### Immunohistochemical staining

Immunohistochemical staining was performed according to the procedure described in our previous studies. Renal tissue was fixed in 4.5% buffered formalin, dehydrated, and embedded in paraffin. For general histology, sections were stained with Masson's trichrome stain.

For immunofluorescent staining, primary antibodies and fluorescent-conjugated secondary antibodies were applied to the sections. For assessment of renal fibrosis, Masson trichrome staining was performed according to the protocol provided by the manufacturer (Sigma, St. Louis, MO). The average ratio to each microscopic field (200×) was calculated and graphed.

### Immunoblot analysis

Immunoblot analysis of tissue samples was conducted as described previously (Liu et al., 2015b). The densitometry analysis of immunoblot results was conducted with NIH Image software (National Institutes of Health, Bethesda, MD).

### Statistical analysis

All the experiments were conducted at least three times with six samples from different animals. Data depicted in graphs represent the means ± S.E.M. for each group. Inter-group comparisons were made using one-way analysis of variance (ANOVA). Multiple means were compared using Tukey’s test. The differences between the two groups were determined by Student’s t-test. The statistical significant difference between mean values was marked in each graph. *p* < 0.05 is considered significant.

## Results

### PP1 attenuates urine microalbumin and improves renal function in HN rat models

**FIGURE 1:**
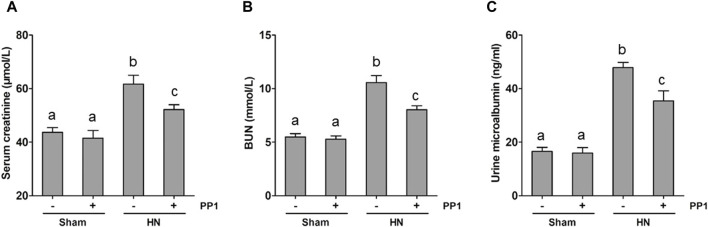
PP1 decreases the progression of proteinuria and improves renal function in HN rats. Serum creatinine **(A)** and BUN **(B)** levels, as well as urine microalbumin **(C)** levels, were examined using an automatic biochemistry assay. **(C)**. Data are represented as the mean ± S.E.M. (*n* = 6). Bars with different superscript letters (a–c) are significantly different from one another (*p* < 0.05).

We employed a rat model of HN induced by oral injection of adenine and potassium oxonate daily to evaluate the impact of PP1, a specific inhibitor of Src, on HN. As shown in Figure 1, the levels of BUN (Figure 1A), serum creatine (Figure 1B), and urine microalbumin (Figure 1C) significantly increased in the HN rat model; PP1 treatment significantly improved the renal function and reduced urine microalbumin. These results indicate that HN was successfully made by daily oral administration of a mixture of adenine and potassium oxonate and suggest that Src is involved in the development of HN.

### Src is activated in the kidney of HN rat models

**FIGURE 2:**
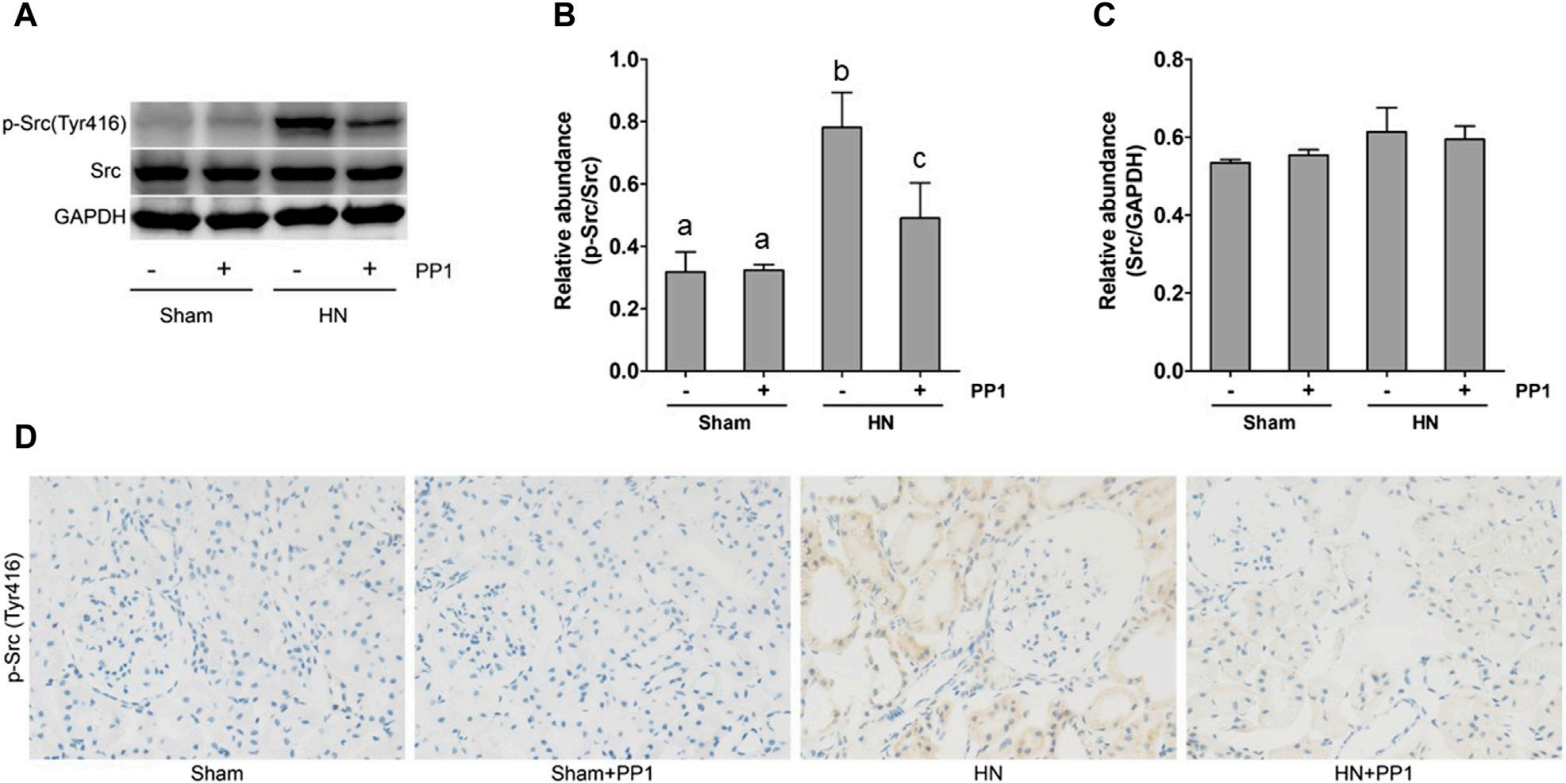
Src is activated in the kidney of HN rats. **(A)** Kidney tissue lysates were subjected to immunoblot analysis with specific antibodies against p-Src, Src, and glyceraldehyde 3-phosphate dehydrogenase (GAPDH). **(B)** The expression level of p-Src was quantified by densitometry and normalized with Src. **(C)** The expression level of Src was quantified by densitometry and normalized with GAPDH. **(D)** Photomicrographs illustrate immunohistochemical staining of P-Src (yellow) in the submesothelial compact zone. Data are represented as the mean ± S.E.M. (*n* = 6). Bars with different superscript letters (a–c) are significantly different from one another (*p* < 0.05).

As a first step to understanding the pathologic role of Src in HN, we investigated the expression of Src in the kidney tissue of HN using immunoblot analysis and immunohistochemical labeling. As seen in Figure 2A, the levels of p-Src (Figure 2B) were significantly increased in the HN rat model compared with the Sham group. Total Src expression levels in the HN rat model were the same before and after PP1 therapy (Figure 2C). PP1 treatment was effective in inhibiting the phosphorylation of Src in HN rat models. Immunohistochemical staining showed that p-Src was minimal in the normal kidney but highly expressed in the renal tubular cells of the HN model (Figure 2D). PP1 also suppressed p-Src expression. These results suggest that Src signaling is active in the HN kidney and sensitive to the PP1 treatment.

### Blockade of Src prevents renal injury and renal fibrosis induced by hyperuricemia in rats

**FIGURE 3:**
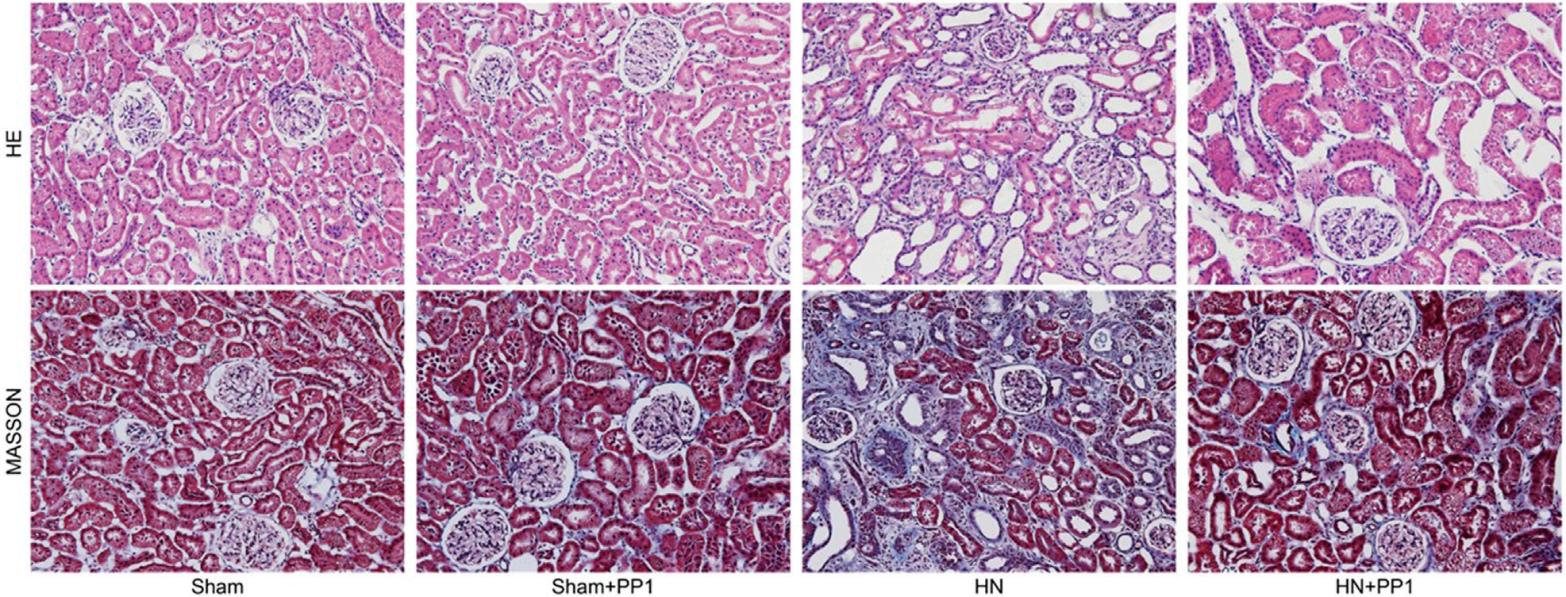
PP1 inhibits the deposition of extracellular matrix in the kidney of HN rats. Photomicrographs illustrating HE and Masson trichrome staining of kidney tissue collected on day 21 after feeding of the mixture of adenine and potassium oxonate with or without PP1.

We proceeded to examine the effect of p-Src inhibition on the development of HN in the rat models. HE staining (Figure 3) of renal tissues from rat models after oral administration of a mixture of adenine and potassium oxonate for 3 weeks displayed dilatation of renal tubules and infiltration of inflammatory cells; Masson’s trichrome staining (Figure 3) revealed substantial interstitial fibrosis. Treatment with PP1 alleviated renal injury and renal fibrosis. These results suggest that PP1 treatment can reduce renal fibrosis brought on by HUA.

### Blockade of Src inhibits the expression of collagen 1 and fibronectin induced by HN in the kidney of rats

**FIGURE 4:**
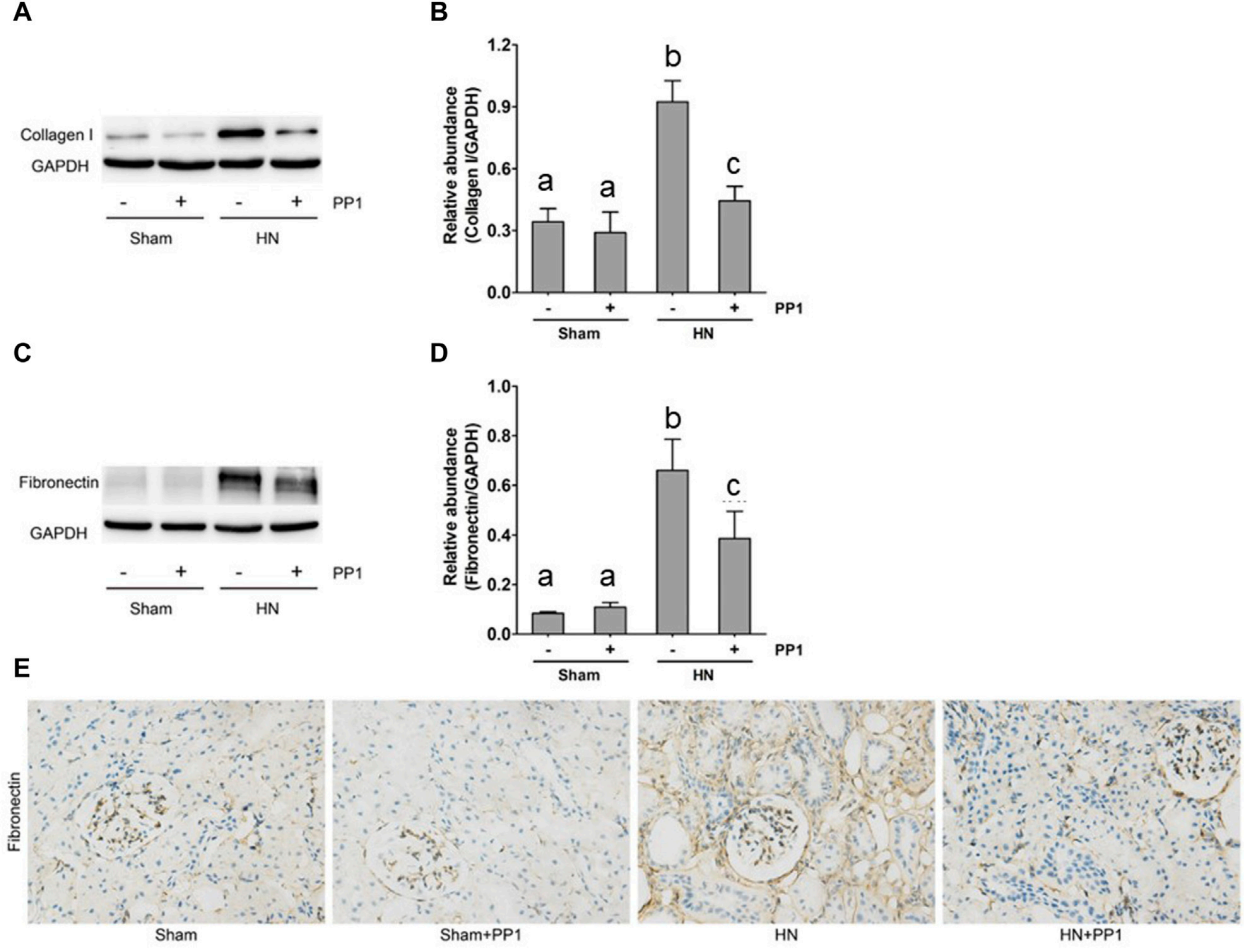
PP1 attenuates the development of renal fibrosis in the HN rat model. Kidney tissue was collected at 21 days after oral administration of a mixture of adenine and potassium oxonate with or without administration of PP1. **(A,C)** The renal tissue lysates were subjected to immunoblot analysis with specific antibodies against collagen 1, fibronectin, or GAPDH. Expression levels of collagen 1 **(B)** and fibronectin **(D)** were quantified by densitometry and normalized with GAPDH, respectively. **(E)** Photomicrographs illustrate immunohistochemical staining of fibronectin (yellow). Data are represented as the mean ± S.E.M. (*n* = 6). Bars with different superscript letters (a–c) are significantly different from one another (*p* < 0.05).

To further study the efficacy of PPT in treating renal fibrosis, we examined the renal expression of collagen 1 and fibronectin, two markers of renal fibrosis via immunoblot analysis in the HN rat model. As shown in Figure 4, the expression of collagen 1 (Figures 4A, B) and fibronectin (Figures 4C, D) increased in the kidney tissue compared with the sham group, and PP1 treatment significantly inhibited this response. Fibronectin immunostaining (Figure 4E) of kidney sections revealed the same results, which were inhibited by PP1 treatment.

### Blockade of Src protects against renal injury and fibrosis by inhibiting the TGF-β1/Smad3 signaling pathway in the kidney of HN rats

**FIGURE 5:**
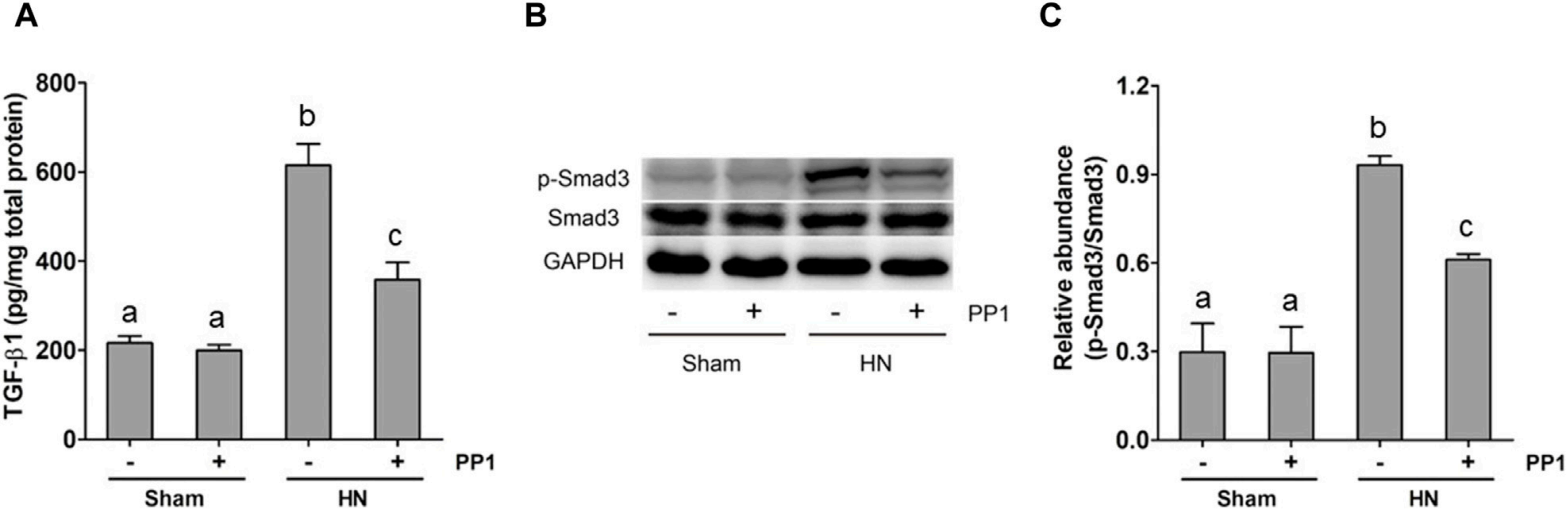
PP1 inhibits the expression of TGF-β1 and the activation of Smad3 in the kidney of HN rats. Renal tissues were collected at 21 days after oral administration of a mixture of adenine and potassium oxonate with or without the administration of PP1. Renal tissue lysates were subjected to ELISA analysis with specific antibodies against TGF-β1 **(A)** and immunoblot analysis against p-Smad3 and Smad3 or GAPDH **(B)**. Expression levels of p-Smad3 were quantified by densitometry and normalized with Smad3 **(C)**. Data are represented as the mean ± S.E.M. (*n* = 6). Bars with different superscript letters (a–c) are significantly different from one another (*p* < 0.05).

The activation of the TGF-β1/Smad3 signaling pathway is crucial to the development of renal fibrosis (Yu et al., 2022). To understand whether Src mediates uric acid-induced activation of TGF-β1 signaling, we analyzed the effect of Src inhibition on the expression of TGF-β1 and phosphorylation of Smads in the rat model of HN. As shown in Figure 5A, the levels of TGF-β1 in the kidney tissue of the HN rat model significantly increased compared with the sham group, as measured by ELISA. Figures 5B, C shows that Smad3 phosphorylation was upregulated in the HN rat model compared with the sham group. Treatment with PP1 reduced levels of TGF-β1 and p-smad3 in the HN rat model. These results indicate that Src functions upstream of the TGF-β1/Smad3 signaling pathway to mediate its activation.

### Blockade of Src inhibits the NF-κB and STAT3 signaling pathways in the kidney of HN rats

**FIGURE 6:**
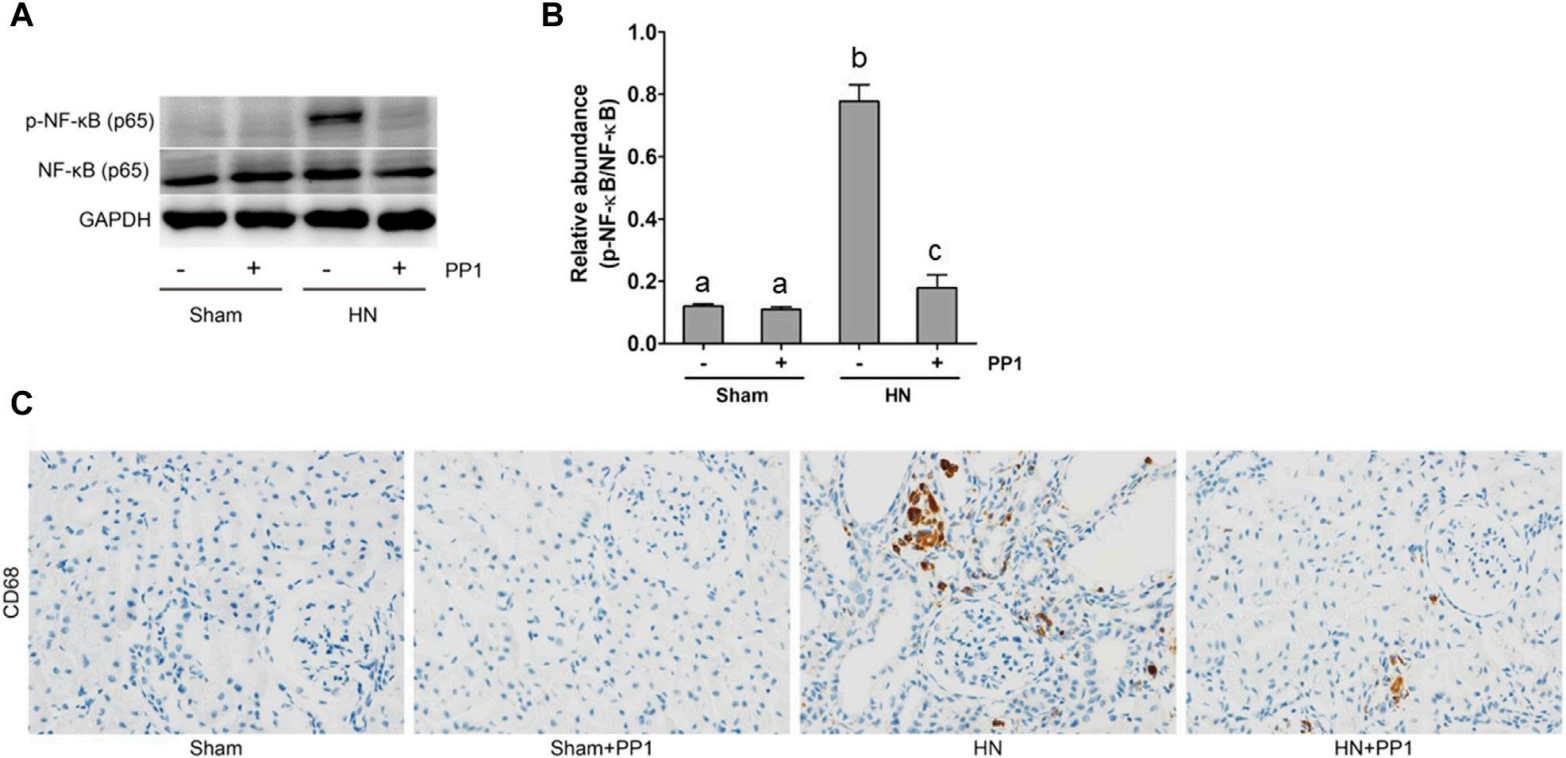
PP1 protects against renal injury and fibrosis by inhibiting the NF-κB signaling pathway in HN rats. Renal tissues were collected at 21 days after the oral administration of a mixture of adenine and potassium oxonate with or without administration of PP1. **(A)** Renal tissue lysates were subjected to immunoblot analysis with specific antibodies against p-NF-κB, NF-κB, or GAPDH. **(B)** Expression levels of p-NF-κB were quantified by densitometry and normalized with NK-κB. **(C)** Immunohistochemistry of CD68-positive macrophages. Bars with different superscript letters (a–c) are significantly different from one another (*p* < 0.05).

**FIGURE 7:**
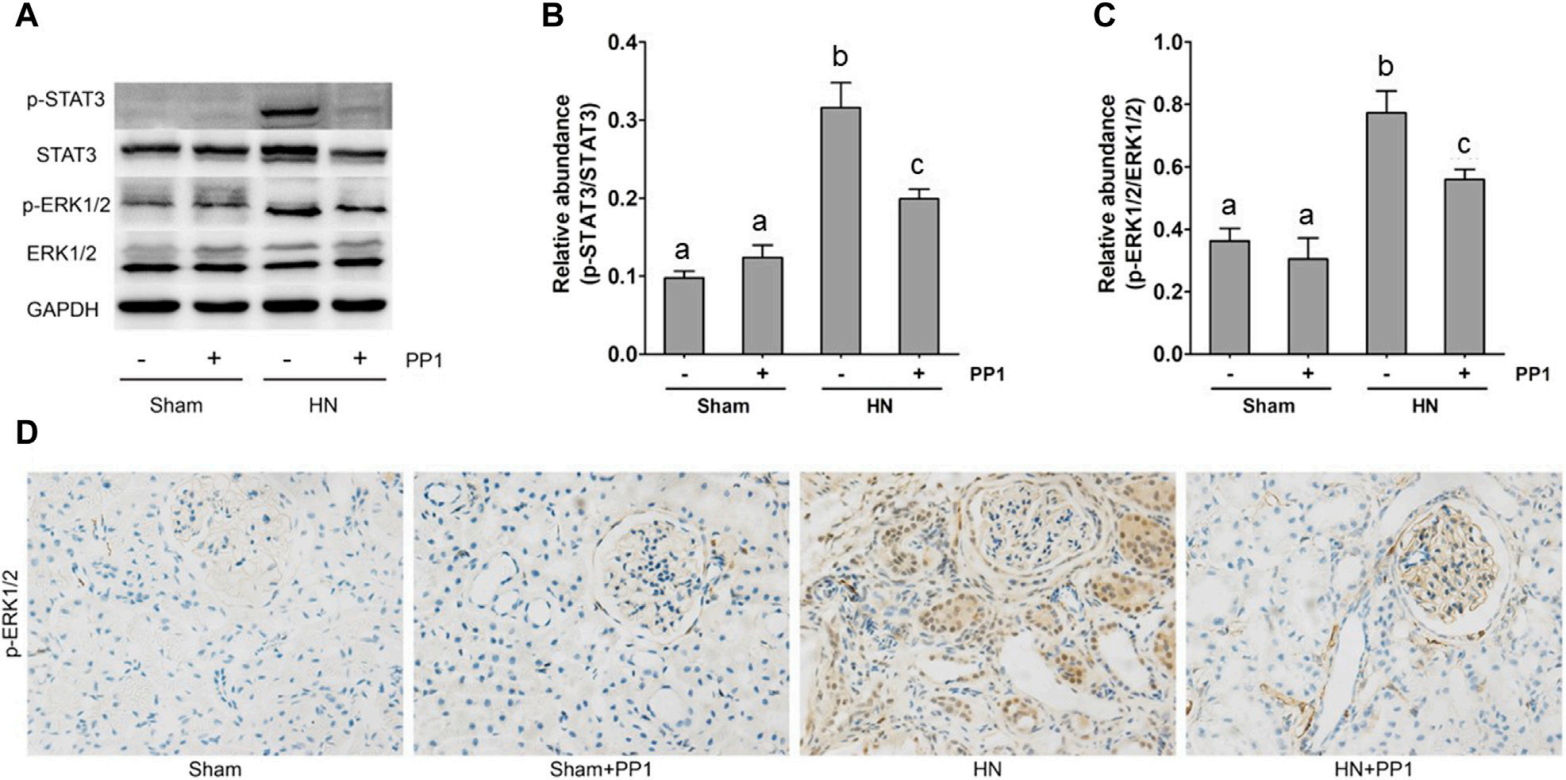
PP1 protects against renal injury and fibrosis through inhibiting the STAT3/ERK1/2 signaling pathway in HN rats. Renal tissues were collected at 21 days after oral administration of a mixture of adenine and potassium oxonate with or without the administration of PP1. **(A)** Renal tissue lysates were subjected to immunoblot analysis with specific antibodies against p-STAT3, STAT3, p-ERK1/2, ERK1/2, and GAPDH. **(B)** The expression level of p-STAT3 was quantified by densitometry and normalized with STAT3. **(C)** The expression level of p-ERK1/2 was quantified by densitometry and normalized with ERK1/2. **(D)** Immunohistochemistry of p-ERK1/2-positive cells. Data are represented as the means ± S.E.M. (*n* = 6). Bars with different superscript letters (a–c) are significantly different from one another (*p* < 0.05).

NF-κB is a pivotal transcription factor that regulates chemokine expression and proinflammatory responses (Liu et al., 2017). Immunoblot analysis of whole kidney lysates showed that expression of phosphorylated NF-kB, STAT3, and ERK1/2 increased in the kidney of HN rats (Figures 6A, B) and inhibition of Src significantly reduced their expressions. p-NF-κB and pSTAT3 were not detectable in the kidney of sham groups both treated and untreated with PP1. The total NF-κB and ERK1/2 levels were not changed in the kidneys of each group of animals (Figures 6A, B). It is well-known that the influx of inflammatory cells into the interstitium is a common pathologic feature of almost all kinds of CKD, including HN. The advancement of uric acid nephropathy is strongly correlated with immune cell infiltration, particularly macrophages. Using an antibody against CD68, a biomarker of active macrophages, we carried out immunohistochemical staining to determine the impact of Src activation on this process. As shown in Figure 6C, the number of CD68-positive macrophages in the injured kidney was remarkably increased in HN rats compared with sham-operated animals, and administration of PP1 significantly reduced their infiltration (Figure 6C). Overall, our results indicate that Src activity contributes to the activation of the NF-κB/STAT3, ERK1/2 (Figures 7A, D) signaling pathway. Treatment with PP1 prevents HN by inhibiting the activation of NF-κB/STAT3, ERK1/2 signaling and macrophage filtration in renal tissue.

### Blockade of Src inhibits the expression of inflammatory cytokines in the kidney of HN rats

**FIGURE 8:**
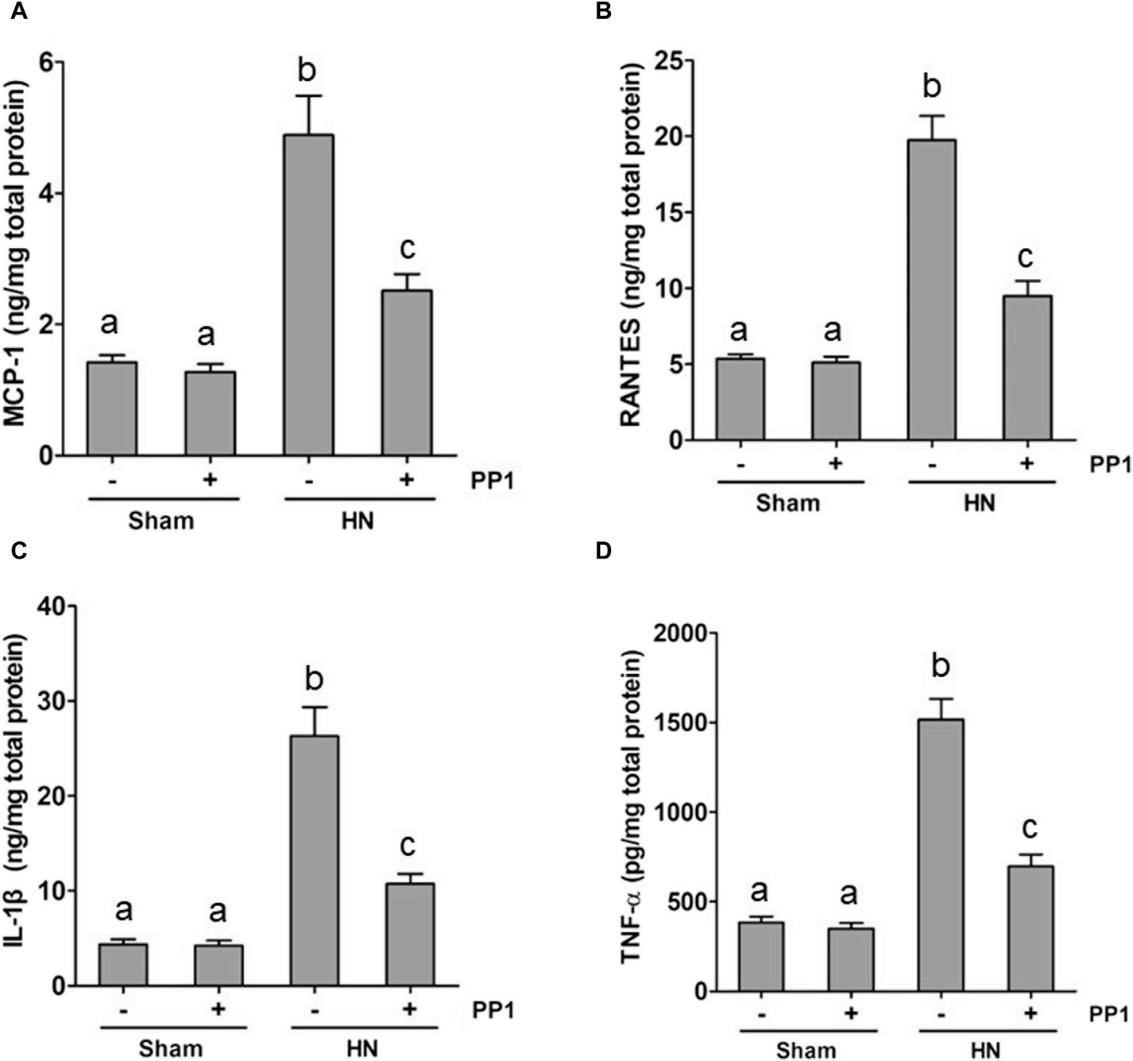
PP1 suppresses the production of multiple proinflammatory cytokines/chemokines in the kidney of HN rats. Renal tissues were collected at 21 days after oral administration of a mixture of adenine and potassium oxonate with or without the administration of PP1. Graphs show the expression levels of MCP-1 **(A)**, RANTES **(B)**, IL-1β **(C)**, and TNF-α **(D)** by ELISA. Data are represented as the means ± S.E.M. (*n* = 6). Bars with different superscript letters (a–c) are significantly different from one another (*p* < 0.05).

Inflammation induced by uric acid is a characteristic feature in the HN rat model, and we thus examined the renal levels of proinflammatory cytokines in the HN rat model and the effect of Src inhibition. Figures 8A–D demonstrate increased levels of many proinflammatory cytokines, including monocyte chemoattractant protein-1 (MCP-1), RANTES, and TNF-α, in the HN rat models relative to those in the control group. Treatment with PP1 significantly decreased the levels of all those cytokines. These results suggest that inhibition of Src suppresses systemic proinflammation induced by uric acid in HN.

### Blockade of Src reduces the uric acid and superoxide dismutase (SOD) activity in the kidney of HN rats

**FIGURE 9:**
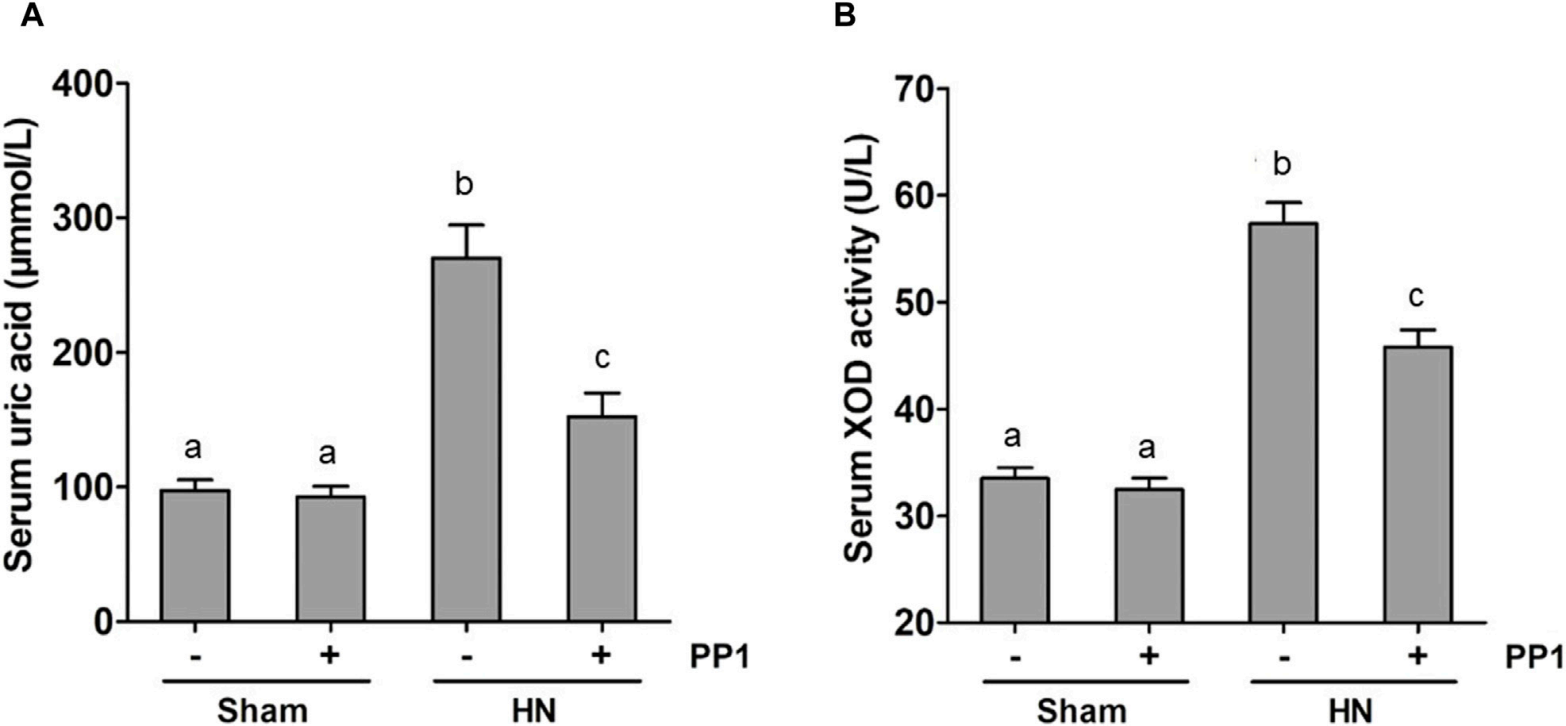
PP1 reduces the serum uric acid and SOD activity in the kidney of HN rats. Blood samples were collected at 21 days after oral administration of a mixture of adenine and potassium oxonate with or without the administration of PP1. Graphs show the expression level of serum uric acid **(A)** and serum SOD activity **(B)**. Data are represented as the means ± S.E.M. (*n* = 6). Bars with different superscript letters (a–c) are significantly different from one another (*p* < 0.05).

Uric acid is the product of purine metabolism, and its increased levels and oxidative stress result in HUA (Wang and Bao, 2013; Xu et al., 2017; Hu et al., 2022), while superoxide dismutase (SOD) is an important member of the antioxidant enzyme system in biological systems, widely distributed in microorganisms, plants, and animals (Atiya et al., 2023). To understand whether Src would mediate the pathogenesis of HN via regulating uric acid and SOD activity, we examined the effect of Src inhibition on the production of uric acid and the activity of serum SOD in HN rats. The serum uric acid level in rates fed with the mixture of adenine and potassium oxonate for 3 weeks was three times higher than in rates that did not receive the mixture (Figure 9A). PP1 treatment significantly reduced serum uric acid levels, which was consistent with the effect of Src inhibition on serum creatinine and BUN levels (Figures 2A, B). Figure 9B shows that the activity of serum SOD nearly doubled in the kidneys of hyperuricemic rats compared with that of the sham group. Administration of PP1 was effective in reducing the activity of serum SOD in hyperuricemic rats. Thus, we suggest that Src contributes in upregulating serum uric acid and SOD activity levels via regulation of the metabolism of uric acid, and PP1 can effectively depress the uric acid and SOD activity in HN rat models.

## Discussion

SFKs have been reported to mediate renal damage and fibrosis in several studies (Seo et al., 2016; Mkaddem et al., 2017; Li et al., 2022). However, it remains unknown whether SFKs play a role in HN. In this study, we demonstrated that (i) Src kinase is activated in renal tissues in rats orally administrated by a mixture of adenine and potassium oxonate; (ii) pharmacological inhibition of SFK with PP1 improves renal function and reduces urine microalbumin at the dose used to reduce the Src phosphorylation; (iii) PP1 lowers renal fibroblast activation and reduces the accumulation of collagen 1 and fibronectin and inhibited the expression of TGF-β1 and phosphorylation of Smad3 and NK-κB in the kidney of rat HN models; and (iv) PP1 inhibits the expression of proinflammatory cytokines in HN. To the best of our knowledge, this is the first study to demonstrate the beneficial effects of PP1 on the amelioration of chronic kidney injury caused by HN.

Like CKD caused by other etiologies, HN is pathologically characterized by the activation and proliferation of renal interstitial fibroblasts, as well as the deposition of ECM components and chronic inflammatory responses (Xiao et al., 2018). So far, the underlying mechanism of HN is largely unknown. Accumulated data indicated that receptor tyrosine kinases (RTKs), such as EGFR, PDGFR, FGFR, and VEGFR, and non-receptor RTKs play a critical role in the pathogenesis of renal fibrosis (Liu et al., 2015b; Liu and Zhuang, 2016; Wang et al., 2016). Among those RTKs, EGFR has been shown to mediate the pathogenesis of HN via activation of TGF-β1 signaling and inflammation and increase in uric acid accumulation in the body; however, it remains unclear whether other growth factor receptors and any non-receptor-RTKs contribute to the development of HN. In this study, by using a rat model of HN that is created by feeding a mixture of adenine and potassium oxonate, we found that administration of PP1, a highly selective Src family kinase inhibitor, improves renal function, attenuates the pathological changes and deposition of ECM proteins, and reduces proinflammatory responses, suggesting the importance of Src in mediating the development and progression of HN.

The underlying mechanism by which Src contributes to HN remains obscure and is being investigated. We observed that PP1 treatment was able to inhibit the expression of TGF-β1 and phosphorylation of Smad3, STAT3, NF-kB, and ERK1/2 at the dose that suppressed Src phosphorylation in the kidney of the HN rat model, suggesting that PP1-elicited suppression of Src activation attenuates the progression of HN at least through the mechanism associated with activation of multiple fibrotic signaling pathways. Previous studies have reported that Src can promote TGF-β1 expression and subsequent activation of Smad3 via transactivation of EGFR and activation of ERK1/2 (Chen et al., 2012). Given that the TGF-β1/Smad3 signaling pathway is required for the activation of renal interstitial fibroblasts and transformation of renal epithelial cells to a mesenchymal phenotype, Src may contribute to the pathogenesis of HN through inducing activation of renal interstitial fibroblasts and epithelial–mesenchymal transition.

STAT3 and NF-kB are two transcriptional factors that are primarily coupled to the production of proinflammatory cytokines and chemokines. Numerous studies have shown that inflammation cytokines and associated molecules are involved in the initiation and progression of HN (Liu et al., 2015a; Yu et al., 2015). In this study, we revealed that blockade of SFK with PP1 reduced expression of multiple cytokines and chemokines such as IL-1β, TNF-α, MCP-1, and RANTES, suggesting that SFK may induce expression of these proinflammatory cytokines and chemokines through activation of STAT3 and NF-kB. In addition, SFKs are required for the activation of different types of inflammatory cells such as neutrophils, monocytes, and macrophages (Okutani et al., 2006). In this context, Tang et al. have recently reported that Src mediates the macrophage–myofibroblast transition (MMT) during UUO-induced renal fibrosis (Tang et al., 2018). In particular, they revealed that Src is a direct Smad3 target gene and acts as the key regulator of TGF-β1-mediated MMT (Tang et al., 2018). On this basis, suppression of Src-mediated proinflammatory responses may be another key mechanism by which PP1 attenuates the pathogenesis of HN.

Uric acid is the product of purine metabolism, and its increased levels result in HUA. Although the role of uric acid levels in HN remains controversial, a high level of uric acid causes a variety of pathological responses, such as oxidative stress, mitochondrial dysfunction, apoptosis, and inflammation. Chronic uric acid injury to the kidney can lead to hyperuricemic nephrology characterized by renal tubular damage, interstitial fibrosis, glomerulosclerosis, and urate crystal deposition. As such, reducing uric acid levels in HN would be another mechanism to ameliorate HN. This study examined the effect of SFK inhibition on serum uric acid levels and illustrated a significantly lower level of uric acid in HN rats subjected to PP1 treatment relative to the rats without PP1 administration. This suggests that inhibition of SFKs may also contribute in attenuating chronic kidney injury by uric acid reduction.

Accumulated evidence reveals that SFKs are involved in the pathogenesis of various fibrotic chronic diseases (Liu et al., 2017). SFKs are activated by multiple profibrotic cytokines, including TGF-β1, EGF, and platelet-derived growth factor (PDGF) (Liu and Zhuang, 2016; Liu et al., 2017; Liu et al., 2019), and are required for multiple cellular processes leading to tissue fibrosis. Here, we showed that high levels of uric acid can induce activation (phosphorylation) of Src in the kidney of the HN rat model, and Src blockade with PP1 is effective in reducing various pathological changes in HN, making it a promising molecular target for the treatment of this disease. Nevertheless, there are some limitations to this study. First, the study was only conducted in animal models, and the results obtained may not be directly translated to patients. Second, the long-term effects and safety profile of PP1 are not clear yet. It is necessary to perform additional studies to assess the potential side effects of PP1 and other SFK inhibitors in animal models. Third, PP1 is a selective inhibitor for several SFKs. The isoform specific inhibitor of SFKs or a genetic approach is needed to clarify the role of individual isoforms of SFKs in HN.

In summary, this study demonstrates the importance of SFKs in mediating HUA and HN through the mechanisms associated with the activation of the TGF-β1/Smad3, STAT3, and NK-κB signaling pathways. Given that HUA can not only induce chronic kidney injury but also serve as a risk factor for CKD caused by various etiologies and several SFK inhibitors are in clinical trials of tumor therapy (Martellucci et al., 2020), pharmacological inhibition of SFKs would likely be a potential treatment for HN and other fibrotic kidney diseases.

## Data Availability

The original contributions presented in the study are included in the article/Supplementary Material; further inquiries can be directed to the corresponding author.
